# Spontaneous Hemothorax from Pulmonary Intralobar Sequestration: A Case Report

**DOI:** 10.5811/cpcem.3259

**Published:** 2024-07-31

**Authors:** Clayton Korson, Jasmine Yu, John M. Pester

**Affiliations:** * St. Luke’s University Health Network Emergency Department, Bethlehem, Pennsylvania; † Rocky Vista University College of Osteopathic Medicine, Parker, Colorado

**Keywords:** *hemothorax*, *tension*, *thoracotomy*, *intralobar*, *sequestration*

## Abstract

**Introduction:**

Pulmonary sequestration is a rarely reported phenomenon where aberrant lung tissue exists independently from the rest of the tracheobronchial network. Complications may include hemothorax; however, there is a paucity of descriptions of this condition in the literature.

**Case Report:**

We describe a case of a pulmonary intralobar sequestration resulting in atraumatic tension hemothorax. A 73-year-old woman presented to our facility in extremis and with complaints of acute-onset flank pain. Her evaluation was notable for a large pulmonary sequestration with a presumed, moderate-sized effusion; however, initial review did not reveal an obvious underlying cause for her symptoms. Shortly after her arrival to the emergency department (ED) she experienced a cardiac arrest. On secondary review of her computed tomographic angiography, it was determined that what was previously thought to be a pleural effusion was a large hemothorax. Following this finding, a finger thoracostomy was performed, which resulted in the immediate evacuation of hemothorax. The thoracostomy was then converted into an ED thoracotomy to assess for active hemorrhage with brief return of spontaneous circulation. Prior to proceeding with emergent operative intervention, the patient’s spouse requested that all further resuscitative efforts cease, and the patient was allowed to expire. In a review of the case, it was determined that the patient suffered from cardiac arrest due to a spontaneous hemothorax secondary to a large intralobar pulmonary sequestration.

**Conclusion:**

Pulmonary intralobar sequestration can result in spontaneous hemorrhage with fatal results. Early and correct interpretation of imaging and surgical intervention are crucial in ED management.

CPC-EM Capsule What do we already know about this clinical entity?

*Intralobar pulmonary sequestration (IPS) occurs when lung tissue is perfused by systemic circulation rather than from pulmonary circulation.*

What makes this presentation of disease reportable?

*Although rare, fatal hemorrhage due to IPS can occur. The most common presentations of IPS are cough and recurrent pneumonia.*

What is the major learning point?

*IPS may cause rapid hemorrhage resulting in cardiovascular collapse due to tension hemothora, or exsanguination. Early surgical intervention may be necessary.*

How might this improve emergency medicine practice?

*Clinicians should be aware that fatal hemorrhage can result from IPS. Resuscitative thoracotomy can aid in decompression of tension hemothorax and hemorrhage control.*

## INTRODUCTION

Pulmonary sequestration is a rare abnormality in which non-functional lung tissue exists in isolation from the rest of the tracheobronchial tree and receives an anomalous blood supply from the systemic circulation.^1^ Cough is the most commonly described presenting sign with hemoptysis noted in severe cases; however, most patients are asymptomatic.^1^ ^,^ ^2^ Intralobar pulmonary sequestration (IPS) as a cause of hemothorax is a rarely reported entity.^3^ ^–^ ^6^ We present an uncommon case of IPS complicated by atraumatic hemothorax requiring emergency thoracotomy for stabilization.

## CASE REPORT

A 73-year-old woman presented to our facility with complaints of sudden onset, left-sided flank pain. The pain began shortly prior to arrival. It was sharp in nature and located in the left flank region with initial radiation of pain from her left collar bone down to her pelvis. Later, she described the pain as radiating to the midthoracic spine. Of note, she had a complex past medical history that included transcatheter aortic valve replacement, chronic heart failure with a reduced ejection fraction, left intralobar pulmonary sequestration, coronary artery disease, hypertension, and biventricular pacemaker. She also had chronic osteomyelitis of her sternum status post washout with an antibiotic course.

On arrival, the patient appeared critically ill, diaphoretic, and clammy. Her initial vital signs included a blood pressure of 93/57 millimeters of mercury, oxygen saturation of 90% on room air, respiratory rate of 26 breaths per minute, heart rate of 60 beats per minute, and a temperature of 36.7° Celsius. On physical exam, the patient was ill-appearing, diaphoretic, and uncomfortable. She was alert and oriented and exhibited no neurological deficits. Heart sounds were regular without evidence of rubs or murmurs. Her lungs were clear to auscultation bilaterally. She had 2+ pulses in all four extremities without evidence of bruits, thrills, mottling, or cyanosis. Her capillary refill was less than two seconds. She had no evidence of ecchymoses, deformities, or rashes.

**Image 1.**
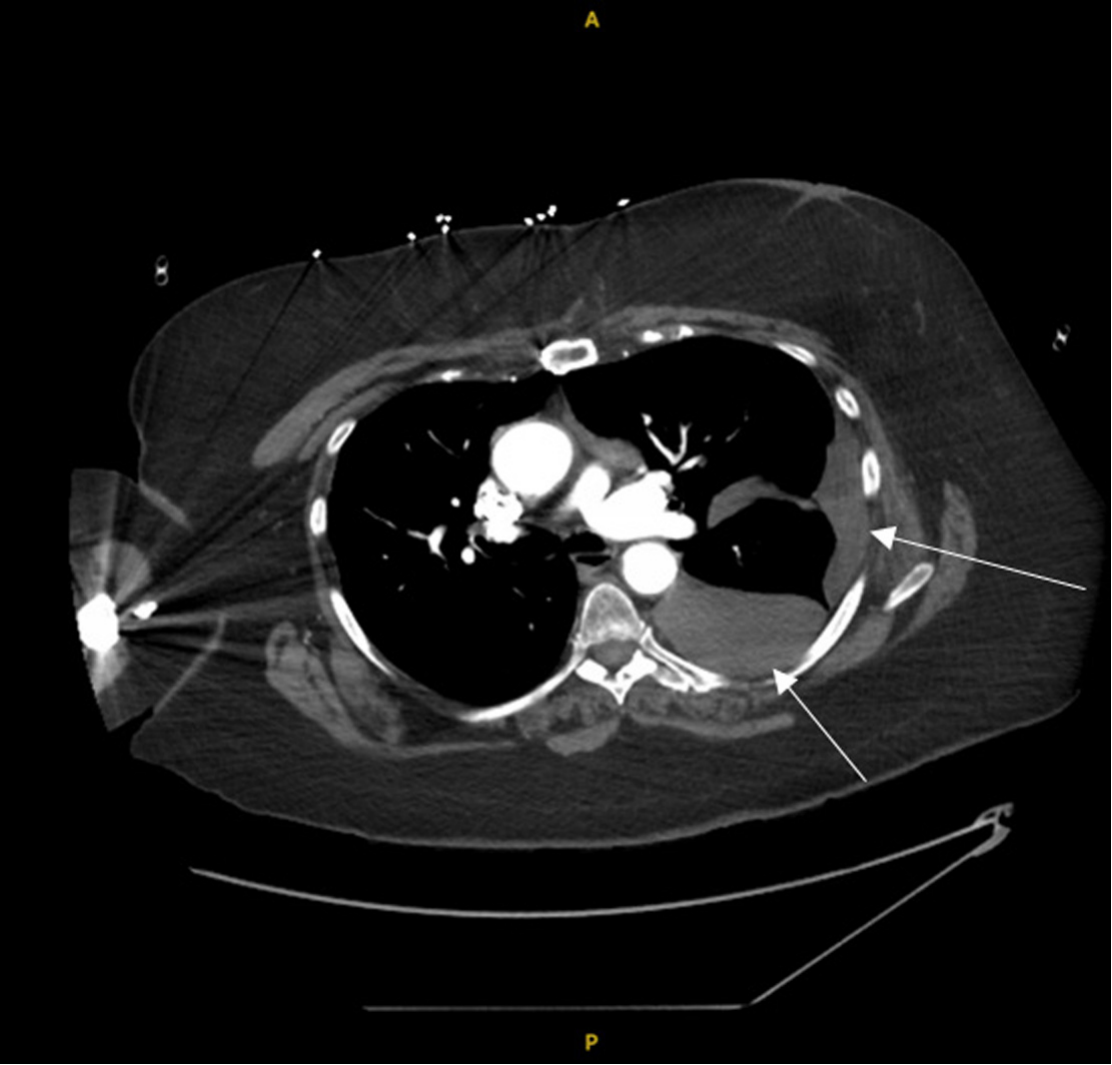
Computed tomography angiography of the chest; axial image showing dense left pleural effusion (arrows).

Immediately after the initial evaluation, a broad evaluation was obtained, which included an emergent computed tomography angiography (CTA) to evaluate for aortic dissection, an electrocardiogram (ECG), troponin, complete blood count, metabolic panel, lipase, and hydromorphone for pain control. Her ECG was notable for a biventricularly paced rhythm, at a rate of 103, with frequent premature ventricular complexes and without evidence of acute ischemia. The CTA was reviewed in real time (Image 1) and showed no evidence of acute aortic pathology.

**Image 2.**
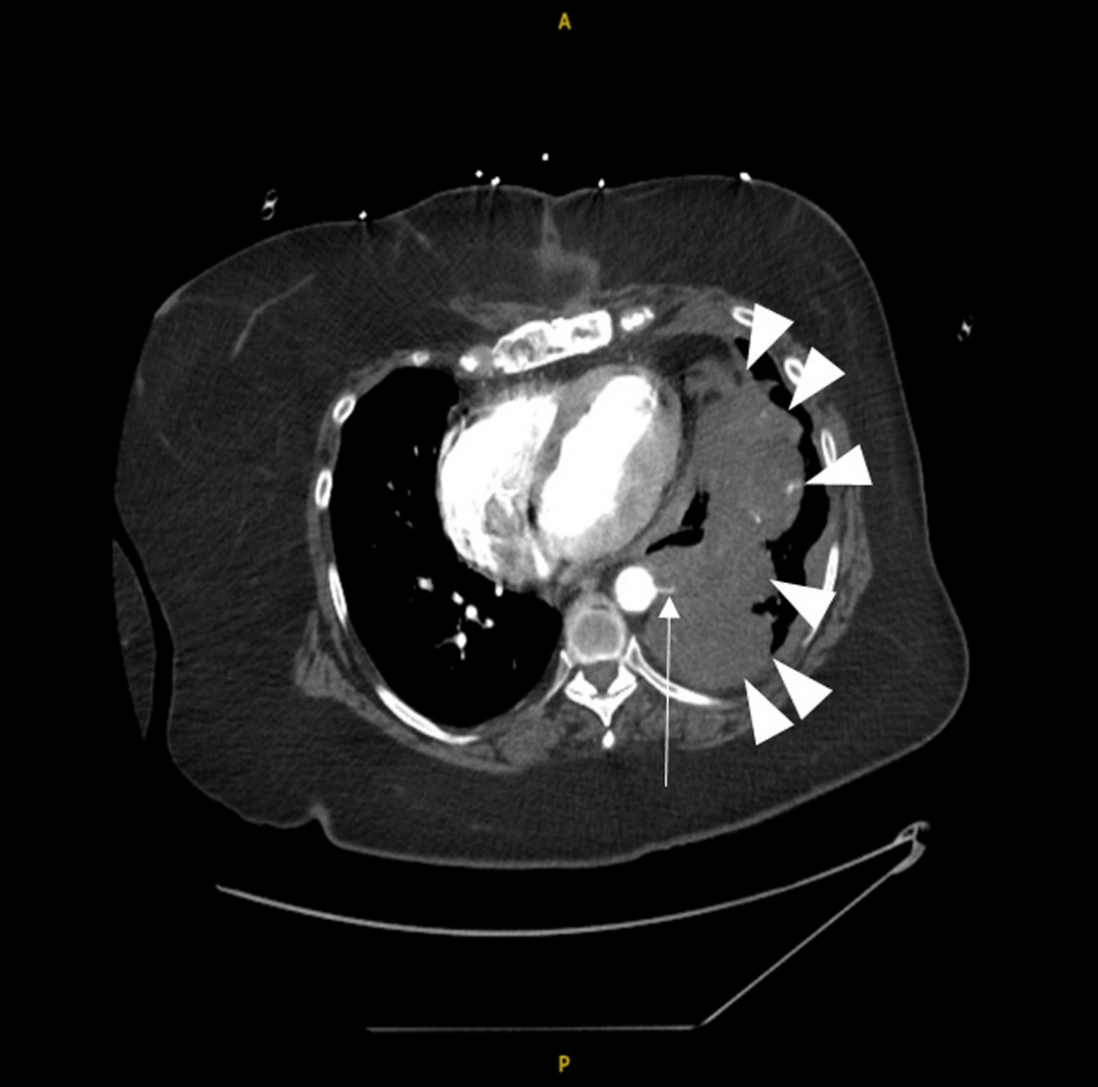
Hyperdensity indicating hemorrhage (triangles) supplied by an artery from a small aortic branch (arrow).

She did have a large, complex, heterogeneous left intralobar mass, consistent with her known pulmonary sequestration, as well as what was presumed to be a moderate, left-sided pleural effusion. Upon reviewing reports from an outside hospital (without the ability to view the actual images), it was deemed that these findings were chronic in nature and unchanged. A delayed sequence CTA was obtained shortly after, which did not show evidence of blush on initial review (Image 2).

Her laboratory workup was notable for a white blood cell count of 12.54 × 10^3^ cells per microliter (μL) (reference range 4.5–11 × 10^3^ cells/μL). hemoglobin of 13.5 grams per deciliter (g/dL) (12.1–15.1 g/dL), blood sugar of 161 milligrams (mg)/dL (70–100 mg/dL), and a high sensitivity troponin of 9 nanograms per liter (ng/L) (0–4 ng/L).

The patient continued to deteriorate, becoming progressively more diaphoretic. Repeat ECG showed a biventricularly paced rhythm, rate of 121, without evidence of acute ischemia. Given her worsening status, cardiology was consulted for possible acute coronary syndrome (ACS) that may have been masked by her ventricularly paced rhythm. While cardiology was in the process of obtaining a transthoracic echocardiogram to assess for regional wall movement abnormalities, the patient went into cardiac arrest. Cardiopulmonary resuscitation was started using standard Advanced Cardiac Life Support (ACLS) protocol. Due to a large volume of emesis, esophageal diversion intubation was performed, followed by endotracheal intubation.

**Image 3.**
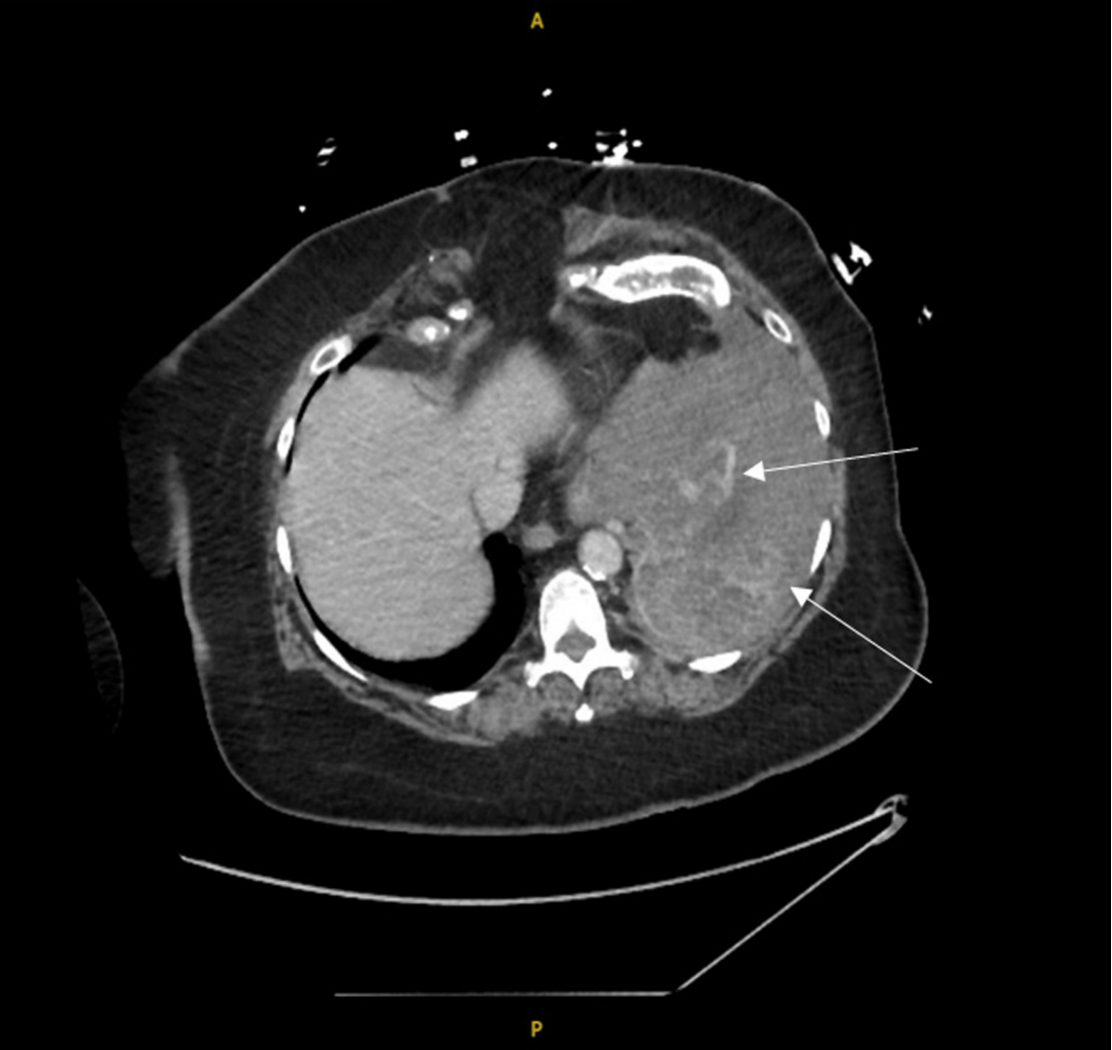
Computed tomography angiography axial image. Delayed sequence imaging demonstrating swirling contrast within the hemothorax, consistent with active hemorrhage (arrows).

The ACLS was continued for approximately 20 minutes without return of spontaneous circulation. Systemic thrombolytics were considered for presumed ACS. Prior to administration of thrombolytic, however, her CTA was reviewed again, and a blush was noted. What was previously thought to be a pleural effusion was actually a large hemothorax with active hemorrhage (Image 3). Given this finding, a finger thoracostomy was performed on the left side, which resulted in the immediate expulsion of a large volume of blood. This procedure was then converted into an emergent thoracotomy to assess for source of hemorrhage and resulted in the evacuation of approximately 500 milliliters of additional blood. A massive transfusion protocol was commenced, acute care surgery arrived at the bedside, and return of spontaneous circulation was achieved.

Prior to proceeding with further surgical intervention, goals of care were discussed with the patient’s husband who wished to cease all further aggressive management. The patient expired thereafter.

In review, it was determined that the patient suffered from cardiac arrest due to a spontaneous, atraumatic hemothorax secondary to a large, hemorrhagic IPS.

## DISCUSSION

Pulmonary sequestration is a rare condition in which a segment of abnormal lung tissue exists with no identifiable tracheobronchial communication and receives its arterial blood supply from the systemic circulation. Pulmonary sequestration is further divided into extralobular and intralobular sequestration. In extralobar pulmonary sequestration, the lung mass is enclosed in its own pleural sac, whereas in IPS the mass lies within the visceral pleura.^1^

Literature review of IPS case reports describe cough and recurrent respiratory infection as the most common presenting symptoms.^2^ In only a few published cases has hemothorax been reported as a complication of IPS, likely caused by spontaneous rupture of an artery feeding the anomalous pulmonary tissue.^3^ ^–^ ^6^ The literature supports the use of computed tomographic angiography for the diagnosis of pulmonary sequestration and demonstration of the arterial supply. Per prior reports, the most common site of intrapulmonary type sequestration is the left lower lobe of the lung, which is consistent with our patient’s findings.^7^ Of the reported cases that describe hemothorax as a rare presentation of IPS, two describe the use of emergency thoracotomy to find the origin of bleeding and to achieve hemostasis in emergent cases with patients presenting with massive intrapulmonary hemorrhage and in shock.^3^ ^,^ ^4^ Following initial stabilization measures, all studies describing IPS complications advocate for resection of the sequestered tissue for prevention of future major medical complications.

## CONCLUSION

This case report describes a rare presentation of intralobar pulmonary sequestration with spontaneous hemothorax resulting in cardiac arrest. Early diagnosis and resection of the abnormal lung tissue should be considered in all patients with findings of pulmonary sequestration. Our case also adds to the literature a rare instance where emergency department thoracotomy should be considered to aid in the stabilization of a medical patient.
